# Diffuse large B-cell lymphoma with cutaneous involvement in a patient with xeroderma pigmentosum type C

**DOI:** 10.1016/j.jdcr.2024.04.039

**Published:** 2024-05-07

**Authors:** Melissa R. Laughter, Cosmin A. Tegla, Shashi Pawar, Ata S. Moshiri, Seth J. Orlow

**Affiliations:** a Ronald O Perelman Department of Dermatology, NYU Grossman School of Medicine, New York, New York; b Division of Hematology and Medical Oncology, NYU Langone Health, New York, New York; c Department of Pathology, NYU Grossman School of Medicine, New York, New York

**Keywords:** diffuse large B cell lymphoma, DNA damage repair, xeroderma pigmentosum type C

## Introduction

Xeroderma pigmentosum type C (XP-C) is a rare autosomal recessive genetic disorder characterized by an inability to repair DNA damage caused by ultraviolet (UV) radiation. Individuals with XP-C have a heightened susceptibility to skin cancer because their genetic mutation hinders the repair of DNA damage induced by exposure to UV radiation.^1^ XP poses a significant health risk to affected individuals, making UV protection and regular screenings vital components of their care.^2^

## Case presentation

**Fig 1:**
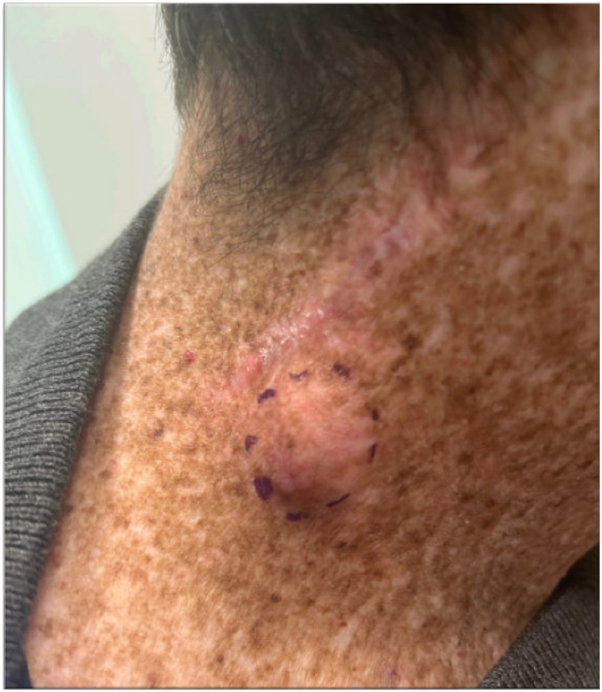
Right lateral neck of patient showing an erythematous nodule along with demarcation of the punch biopsy site.

A 37-year-old male with XP-C, had a notable history of multiple skin cancers, predominantly cutaneous squamous cell carcinoma, necessitating numerous procedures including the removal of an squamous cell carcinoma invasive to the zygoma as a teenager, followed by adjunctive radiation therapy. He presented to his dermatologist with rapid onset of 2 firm subcutaneous nodules, 1 on his right lateral neck (Fig 1) and the other on his left medial thigh. Punch biopsies were performed of each of the nodules.

**Fig 2:**
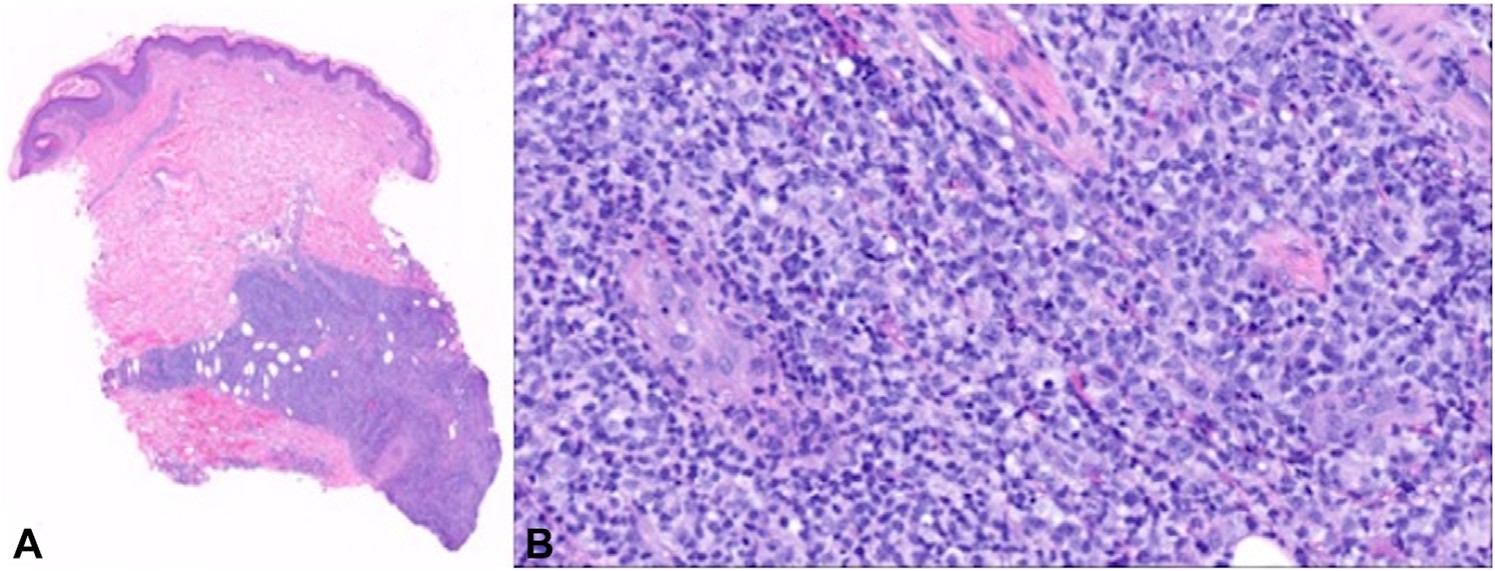
Hematoxylin and eosin (H&E) showing diffuse infiltration of lymphocytes throughout the dermis extending into the subcutaneous tissue (**A**). High power shows the cells to be large, with prominent nucleoli (**B**).

**Fig 3:**
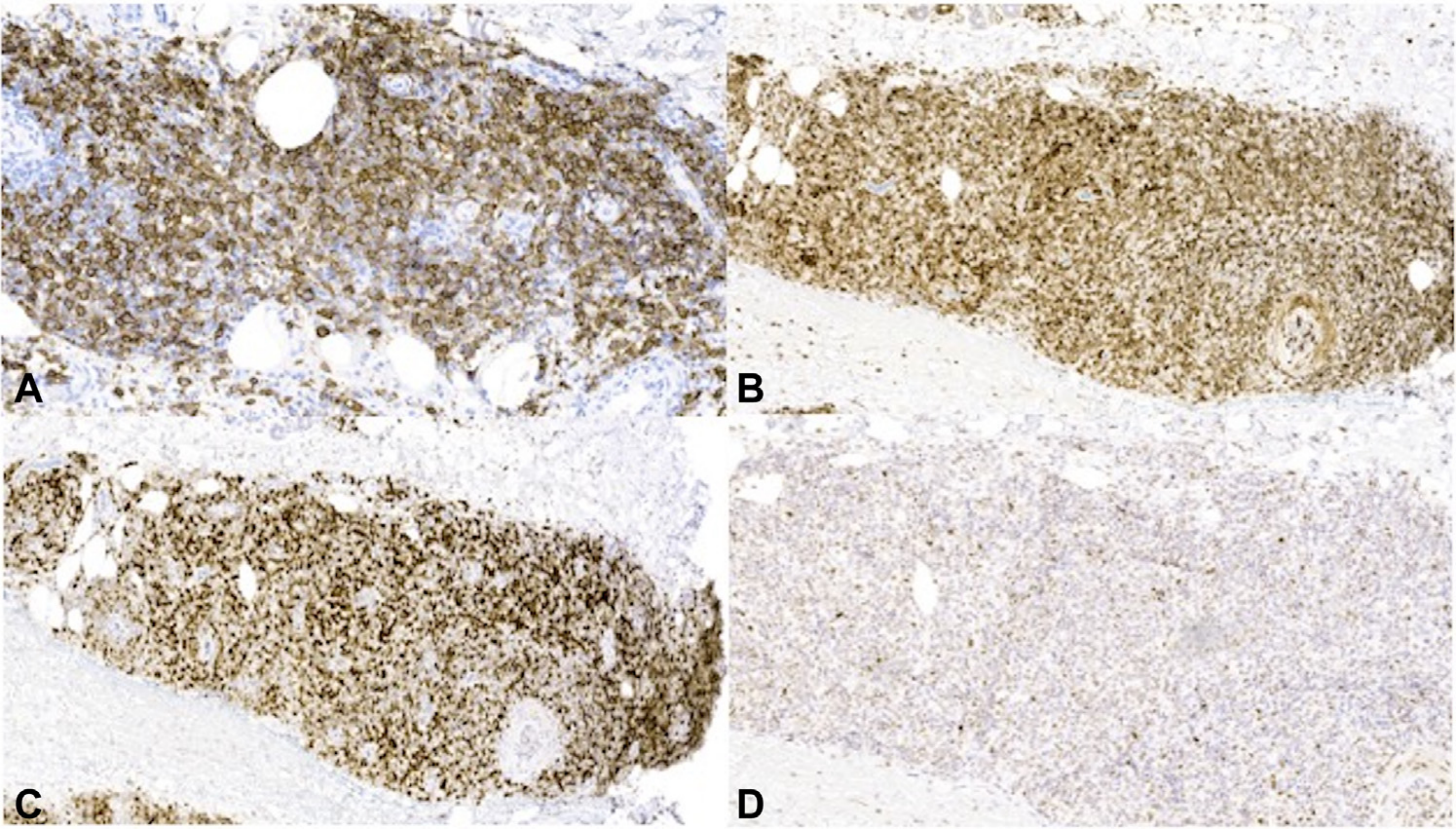
CD20 highlights the larger lymphoid population (**A**), which shows strong and diffuse expression of B-cell lymphoma (BCL)2 (**B**) as well as MUM-1 (**C**), with focal weak expression of BCL6 (**D**).

Microscopic analysis of both nodules uncovered extensive pandermal infiltrates characterized by large, atypical lymphocytes exhibiting numerous mitotic figures, accompanied by prominent foci of necrosis (Fig 2, *A* and *B*). The observed nodules exhibited a notably elevated proliferative index, as indicated by Ki-67 staining (not shown). Immunohistochemical investigations unveiled a distinctive profile, featuring positive staining for CD20, CD79a, BCL-2, and MUM-1, along with patchy and weak positivity for BCL-6 (Fig 3). Molecular studies, specifically examining MYC, BCL6, and BCL2 genes, revealed no rearrangements. Together these studies supported a diagnosis of diffuse large B-cell lymphoma with cutaneous involvement.

The patient underwent further diagnostic workup, including a positron emission tomography-computed tomography scan, which delineated hypermetabolic subcutaneous masses on the right shoulder and left medial thigh, concurrent with right cervical and left axillary lymphadenopathy. Additionally, subcapsular hepatic lesions raised suspicions of systemic involvement. In response to this comprehensive evaluation, the patient commenced chemotherapy with rituximab, cyclophosphamide, doxorubicin, vincristine, and prednisone (R-CHOP) and completed 2 cycles of treatment in total. However, he tolerated poorly R-CHOP chemotherapy, necessitating dose reductions and resulting in treatment delays. Despite these challenges, the patient demonstrated a favorable response to treatment as evidenced by positron emission tomography-computed tomography. Unfortunately, he developed multiple episodes of bacteremia with enteric organisms and pneumonia, ultimately leading to hypoxic respiratory failure and death. It is important to note that despite experiencing grade 4 neutropenia, his absolute neutrophil count fully recovered after each chemotherapy cycle, suggesting that nonhematological complications, such as colitis and bacterial translocation, could have contributed to his persistent bacteremia.

## Discussion

This case underscores the importance of recognizing that XP-C, primarily known for its association with skin cancers due to impaired DNA repair following UV radiation exposure, it may also be linked to lymphoproliferative disorders. A previous case series revealed that germline mutations in the XP-C gene can be associated with various hematologic neoplasms, including myelodysplastic syndrome (MDS), acute leukemias, and high-grade lymphoma. In a cohort of 117 XP patients, 4 individuals with XP-C mutations developed these malignancies, emphasizing the broader spectrum of internal cancers beyond skin cancers traditionally associated with XP.^3^

Another cohort study reported a notably high frequency of hematological malignancies, primarily myeloid, in a subgroup of XP-C patients sharing the same XP-C mutation prevalent in North African families. These patients with MDS and acute myeloid leukemia (AML) displayed unique characteristics, including early age onset, bone marrow dysplasia, low-intermediate blast cell counts, TP53 mutations, and complex karyotypes (del5q and del7q). Additionally, the study highlights a frequent occurrence of TP53 mutations in skin tumors of XP patients, suggesting shared oncogenesis pathways and hypermutability.^4^ The documented predisposition to myeloid cancers in XP-C emphasizes the importance of careful monitoring, particularly through annual blood examinations in delTG XPC homozygous patients. Clinicians are advised to maintain a high suspicion for MDS/AML and consider bone marrow examinations in case of consistent abnormalities in blood counts.

Additionally, a comprehensive study conducted by Yurchenko et al aimed to elucidate the mutational landscape in XP-C patients, with a focus on internal tumors. Whole-genome sequencing of these tumors revealed a mutator phenotype or increased mutation rate in XP-C patients, which may explain their heightened susceptibility to internal cancers, specifically hematological malignancies. The identified XP-C cancers Signature “C” shared a remarkable resemblance with COSMIC Signature 8, previously linked to sporadic tumors and homology-repair deficiency. This signature is now associated with nucleotide excision repair (NER) deficiency in organoid models. Comparative analysis with mutational profiles and Signature “C” from XP-C tumors, XPC, and mouse Ercc1 knockouts underscores dysfunctional NER as a common genetic basis. This multifaceted approach provides valuable insights into the intricate relationship between GG-NER deficiency, mutational processes, and the development of internal tumors in XP-C patients.^5^

In our case, the histological and immunohistochemical findings point towards a rare but sometime aggressive lymphoma, reinforcing the importance of considering lymphoma as a potential diagnosis in XP-C patients presenting with cutaneous lesions. C-MYC, BCL2, and BCL6 genes are the most commonly oncogenes involved in B-cell lymphomas.^6^ Fluorescence in-situ hybridization analysis of this case showed the absence of molecular rearrangements in C-MYC, BCL6, and BCL2 genes.

As mentioned, this patient developed infectious complications following chemotherapy leading to sepsis and eventual death. While the available literature on the subject is limited, it generally indicates that patients with XP can tolerate chemotherapy, despite the presence of a DNA repair gene mutation. XP exhibits a notably lower incidence of bone marrow aplasia when contrasted with other defects in DNA repair mechanisms, including Fanconi anemia, Bloom syndrome, and ataxia telangiectasia.^7^ An instance of a 33-year-old male XP patient diagnosed with acute megakaryoblastic leukemia showed that full doses of AML-type chemotherapy, including allogeneic stem cell transplantation following a conditioning regimen with busulfan and cyclophosphamide, were well-tolerated without unusual toxicity.^8^ However, there have been 4 reported cases of severe adverse effects, all resulting in acute renal failure and fatality, from cisplatin administration in XP patients. A possible explanation for the increased toxicity is that the DNA intrastrand cross-link damage from cisplatin is mainly repaired by the nucleotide excision repair system. The NER pathway involves several proteins, including those encoded by the XPA, XPC, and ERCC2 (XPD) genes, mutations in each of which can cause a form of XP.^9^, ^10^, ^11^ As discussed by Melis et al., NER – and a role for XP-C – have also been implicated in the repair of DNA oxidative damage.^12^ If the inactivity is significant, severe damage to normal cells might not be repaired after cisplatin treatment, potentially leading to fatal consequences. Acute kidney injury is a well-documented, dose-limiting toxicity, and complication of cisplatin administration. This case highlights the potential for severe toxicities and poor tolerance to R-CHOP chemotherapy in patients with XP, underscoring the significance of this report.

Further investigation is required to comprehend fully the impact of chemotherapy in XP patients. It's crucial to remember that a single patient's tolerance to chemotherapy may not be indicative of the broader XP patient population, as individual responses can differ. This report contributes to the limited literature on XP-C-related lymphoproliferative disorders, highlighting the need for continued research and vigilance in the management of XP-C patients.

In conclusion, this case of a 37-year-old male with XP-C developing multifocal diffuse large B-cell lymphoma with cutaneous involvement with a fatal outcome following an initial response to R-CHOP therapy underscores the intriguing link between XP-C and lymphoproliferative disorders. This poignant outcome emphasizes the complexities involved in managing such cases and underscores the ongoing challenges in the field.

## Conflicts of interest

None declared.
